# Bio-inspired heterogeneous composites for broadband vibration mitigation

**DOI:** 10.1038/srep17865

**Published:** 2015-12-08

**Authors:** Yanyu Chen, Lifeng Wang

**Affiliations:** 1 Department of Mechanical Engineering, State University of New York at Stony Brook, Stony Brook, New York 11794, USA

## Abstract

Structural biological materials have developed heterogeneous and hierarchical architectures that are responsible for the outstanding performance to provide protection against environmental threats including static and dynamic loading. Inspired by this observation, this research aims to develop new material and structural concepts for broadband vibration mitigation. The proposed composite materials possess a two-layered heterogeneous architecture where both layers consist of high-volume platelet-shape reinforcements and low-volume matrix, similar to the well-known “brick and mortar” microstructure of biological composites. Using finite element method, we numerically demonstrated that broadband wave attenuation zones can be achieved by tailoring the geometric features of the heterogeneous architecture. We reveal that the resulting broadband attenuation zones are gained by directly superimposing the attenuation zones in each constituent layer. This mechanism is further confirmed by the investigation into the phonon dispersion relation of each layer. Importantly, the broadband wave attenuation capability will be maintained when the mineral platelet orientation is locally manipulated, yet a contrast between the mineral platelet concentrations of the two constituent layers is essential. The findings of this work will provide new opportunities to design heterogeneous composites for broadband vibration mitigation and impact resistance under mechanically challenging environmental conditions.

Periodic structures and materials with spatially modulated elastic constants and densities have attracted intensive research interests due to their capabilities to manipulate the propagation of sound and heat. The introduction of inherent periodicity leads to modification of phonon dispersion relations, providing avenues to tailor group velocities^1^,^2^,^3^. One of the remarkable features in phonon dispersion relations is the existence of complete band gaps: frequency ranges where the propagation of phonon is prohibited irrespective of indecent angles^4^,^5^. The formation of complete band gaps is attributed to Bragg scattering and/or local resonances, which are intrinsically governed by the geometric arrangements and material properties^6^,^7^,^8^,^9^,^10^. In this regard, periodic structures are termed as phononic crystals or acoustic metamaterials, respectively, depending on the physical mechanisms of band gap formation. Phononic crystals and acoustic metamaterials have demonstrated great potential in practical applications, including wave filtering^11^,^12^,^13^, acoustic cloaking^14^, heat management^15^,^16^, and energy harvesting^17^,^18^. Despite the intensive research interests and enticing potential applications, current phononic crystals and acoustic metamaterials still suffer from some drawbacks. For example, conventional phononic crystals and acoustic metamaterials often operate in limited frequency ranges due to the narrow bandwidth of their band gaps. Moreover, the mechanical properties of these materials are often conflicting with the demand for high dissipation of wave energy induced by vibrations and shocks, resulting in the poor performance under mechanically challenging environmental conditions^19^,^20^. These mutually exclusive properties pose great challenges for engineering design to address the competing constraints between vibration mitigation and mechanical properties.

Structural biological materials have long served as a source of inspiration for developing high performance composites. Indeed, many structural biological materials such as seashells, bone, and teeth have developed sophisticated architectures enabling an unusual combination of high stiffness, high strength, fracture toughness, and energy dissipation^21^,^22^,^23^,^24^,^25^,^26^,^27^,^28^. A typical example is nacre, the inner layer of seashell, which is composed of 95 *vol*.% of hard mineral aragonite imbedded within a soft organic matrix, exhibits a fracture toughness about three orders of magnitude higher than that of pure aragonite^29^. In addition, nacreous layer together with a prismatic layer in seashell has developed a multilayered protecting system to maintain their structural integrity under dynamic attack^30^. Notably, such a heterogeneous architecture enables a combination of enhanced stiffness and surface hardness in the upper layer, with flexural modulus and fracture toughness in the inner layer^31^,^32^,^33^. Similar heterogeneous architectures have also been observed in human teeth^34^, consisting outer hard and brittle enamel layer and the relatively soft but tough dentin layer, and fish scale armor^35^,^36^,^37^,^38^, which possess multiple mineralized layers where each layer is composed of a different nanocomposite material with varying structural and mechanical anisotropy. These natural design principles not only reveal the mechanisms responsible for the outstanding mechanical properties of structural biological composites, but also provide us clues to design and develop high performance structural composites.

In this work, we investigate the elastic wave propagation in bio-inspired heterogeneous composites, aiming at addressing the bandwidth limitation and the conflict between vibration mitigation and mechanical performance of conventional periodic structures. The model system under investigation consists of two layers where each layer consists of high volume of platelet-shape reinforcements embedded in the low-volume soft matrix, which is a typical “brick and mortar” microstructure of biological structural composites (see Fig. 1). The vibration mitigation capability of the proposed heterogeneous composites is demonstrated by studying the transmission property of the normally incident elastic wave using the finite element method. Physical mechanisms responsible for the vibration mitigation are revealed. Furthermore the effect of local characteristics of the heterogeneous architecture on the wave attenuation capability is investigated.

## Modeling Methodology

**Figure 1:**
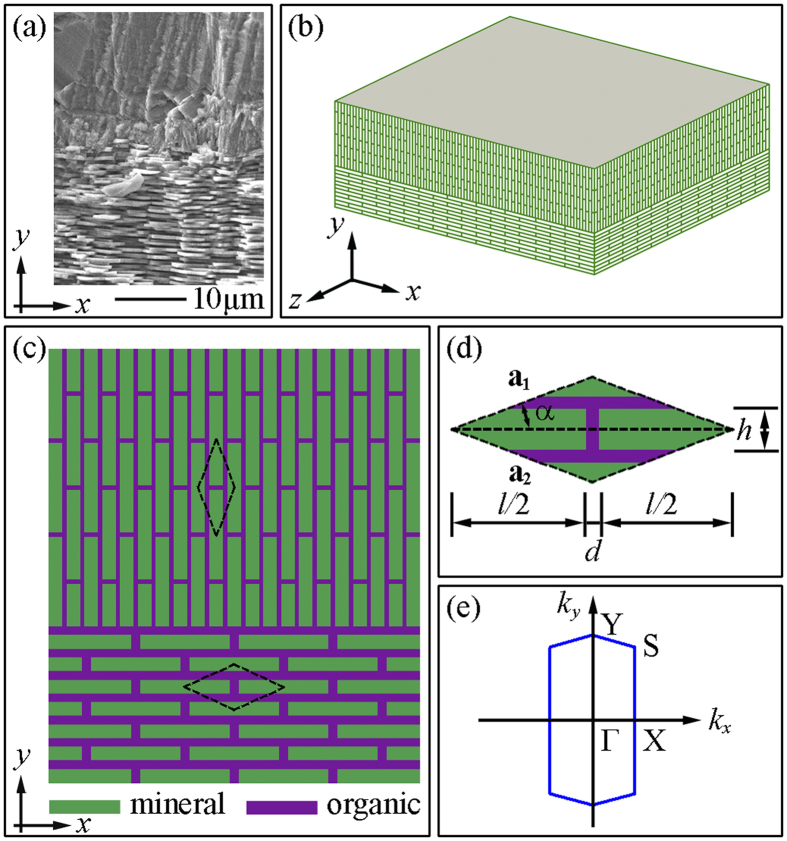
(**a**) A scanning electron microscope image of the cross-section of the prismatic layer and nacreous layer in a *Trochus niloticus* shell. (**b**) Heterogeneous architecture of the proposed composite consists of two layers, where the normal of the layers is perpendicular to the *x-z* plane. The minerals in the upper layer are oriented in the *y*-direction while the mineral platelets in the lower layer are oriented in the *x*-direction. (**c**) Idealized 2D representative of the proposed heterogeneous composite architecture. (**d**) Unit cell for the calculation of the phononic dispersion relation and (e) the corresponding first Brillouin zone. The lattice constants are given by **a**_**1**_ = [(*l+d*)/2, tan*α*∙(*l+d*)/2] and **a**_**2**_ = [(*l+d*)/2, -tan*α*∙(*l+d*)/2], where *l* is the length of the mineral platelet, *d* is the thickness of the organic matrix and *α* is one half of the lattice angle. *h* is the height of mineral platelet. The construction of first Brillouin zone can be found in our previous work^42^.

Fig. 1(c) shows the schematics of the proposed heterogeneous architecture consists of two layers with elastic wave incident normally to the top surface. The periodicity of each layer is characterized by a rhombic lattice with vectors **a**_**1**_ = [(*l+d*)/2, tan*α*∙(*l+d*)/2] and **a**_**2**_ = [(*l+d*)/2, -tan*α*∙(*l+d*)/2], where *l* is the length of the mineral platelet, *d* is the thickness of the organic matrix and *α* is half of the lattice angle (see Fig. 1d). The volume fraction of the mineral platelets is defined as *V*_*f*_  = 2*lh*/[(*l*+*d*)^2^·tan*α*], where *h* is the height of mineral platelet. Here we assume *l* = 10 μm and *d* = 0.2 μm, which are similar to the reported geometric parameters of nacre^39^,^40^,^41^. The lattice angle in each layer is taken as 2*α* = 30°. For these given lattice constants of the unit cell, the first Brillouin zone can be constructed accordingly^42^ (see Fig. 1e). The mineral platelets and the organic matrix are assumed to be isotropic and linearly elastic. Their properties are characterized by, Young’s modulus *E*_*m*_ = 100 GPa, Poisson’s ratio *v*_*m*_ = 0.30, and density *ρ*_*m*_ = 2950 kg/m^3^ for mineral platelets; Young’s modulus *E*_*o*_  = 20 MPa, Poisson’s ratio *v*_*o*_  = 0.30, and density *ρ*_*o*_ = 1350 kg/m^3^ for organic matrix. The transmission spectra and phonon dispersion relations are obtained by conducting frequency domain analyses and eigenfrequency analyses, respectively. More details concerning the numerical modeling can be found in the Methods section.

## Results

### Broadband vibration mitigation

**Figure 2:**
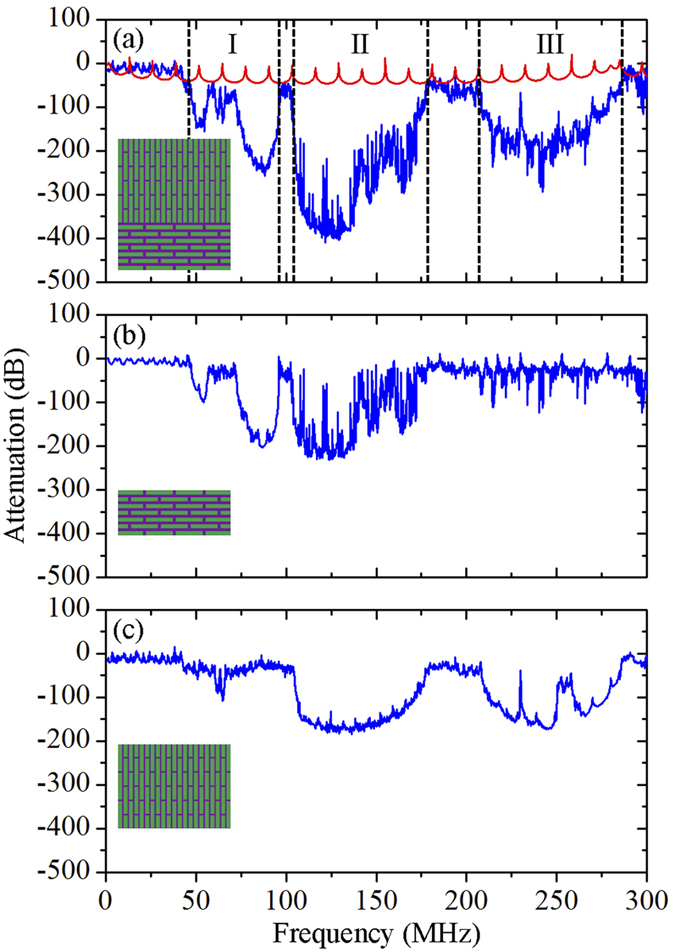
(**a**) Transmission spectrum of the proposed heterogeneous composite (the blue line), which is divided into three regions I, II, and III, and transmission spectrum of the bulk mineral (the red line). (**b**,**c**) Transmission spectrum of the lower layer and upper layer, respectively. The insets show the schematics of the heterogeneous composite and its lower and upper layer.

Figure 2 (a) shows the transmission spectrum of the proposed heterogeneous composite, where the volume fractions of the mineral platelets in the lower layer and the upper layer are set to 0.70 and 0.90, respectively, mimicking those in typical structural biological materials. Multiple attenuation zones within 46 ~ 285 MHz are observed in the transmission spectrum as compared to the transmission spectrum of the bulk mineral. To gain an intuitive understanding of these multiple attenuation zone formations, we calculate the transmission spectrum of each constituent layer, as shown in Fig. 2(b,c). The resulting attenuation zones of the lower layer mostly lie within 46 ~ 179 MHz, while the attenuation zones of the upper layer appear within 104 ~ 285 MHz. Note that the bandwidths of the attenuation zones in each constituent layer are narrower than those of the heterogeneous composite. By comparing the frequency ranges of the attenuation zones in each layer with those of the heterogeneous composite, we believe that the broadband wave attenuation capability results from the direct superimposition of attenuation zones in each constituent layer. To demonstrate this, we divide the broadband wave attenuation zones in transmission spectrum of the heterogeneous composite into three regions, which are regions I (*f* = 46 ~ 96 MHz), II (*f* = 104 ~ 179 MHz), and III (*f* = 207 ~ 285 MHz), respectively. By comparing these regions with the attenuation zones of each constituent layer, we can conclude that region I and III mainly result from the attenuation zones in the lower and upper layer, respectively. However, the enhanced attenuation zones in region II are attributed to the direct superimposition of those in both lower and upper layer. If the frequency is normalized by 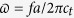 where *a* = 10 μm is the lattice constant and *c*_*t*_  = 75. 5 m/s is the transverse velocity of the matrix phase, the widths of attenuation zones in region I, II, and III are 1.05, 1.58, and 1.64, respectively. These findings indicate that broadband and enhanced wave attenuation capability can be readily achieved by designing the heterogeneous composites with two distinct constituent layers, leading to direct enhancements in vibration mitigation.

**Figure 3:**
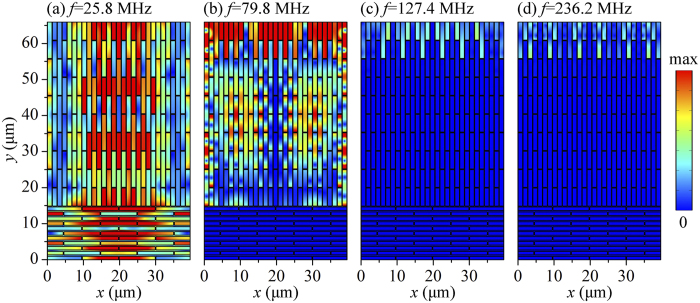
Total displacement field of the proposed heterogeneous composite under different incident wave frequencies. (**a**) *f* = 25.8 MHz, below the attenuation zones, (**b**) *f* = 79.8 MHz, within region I, (**c**) *f* = 127.4 MHz, within region II and (**d**) *f* = 236.2 MHz, within region III. The total displacement is calculated as the magnitude of overall displacement vector with horizontal and vertical components at each material point.

To qualitatively show the broadband wave attenuation capability of the proposed heterogeneous composite, we plot in Fig. 3 the total displacement field of the heterogeneous composite at incident frequencies below the attenuation zones and within regions I, II and III, respectively. At the frequency *f* = 25.8 MHz, below the attenuation zones, the incident wave can pass through the heterogeneous composite without decay. Interestingly, when the incident frequency, *f* = 79.8 MHz, lie within region I, the incident wave passes through the upper layer without decay, whereas it is totally reflected by the lower layer. This observation is consistent with our finding that the attenuation zones within region I are attributed to the lower layer. When the incident frequencies, *f* = 127.4 MHz and *f* = 236.2 MHz, lie within region II and III, respectively, the incident wave is mostly reflected by the upper layer, indicating that the proposed heterogeneous composite can mitigate vibration more effectively.

### Physical mechanisms of the attenuation zones

**Figure 4:**
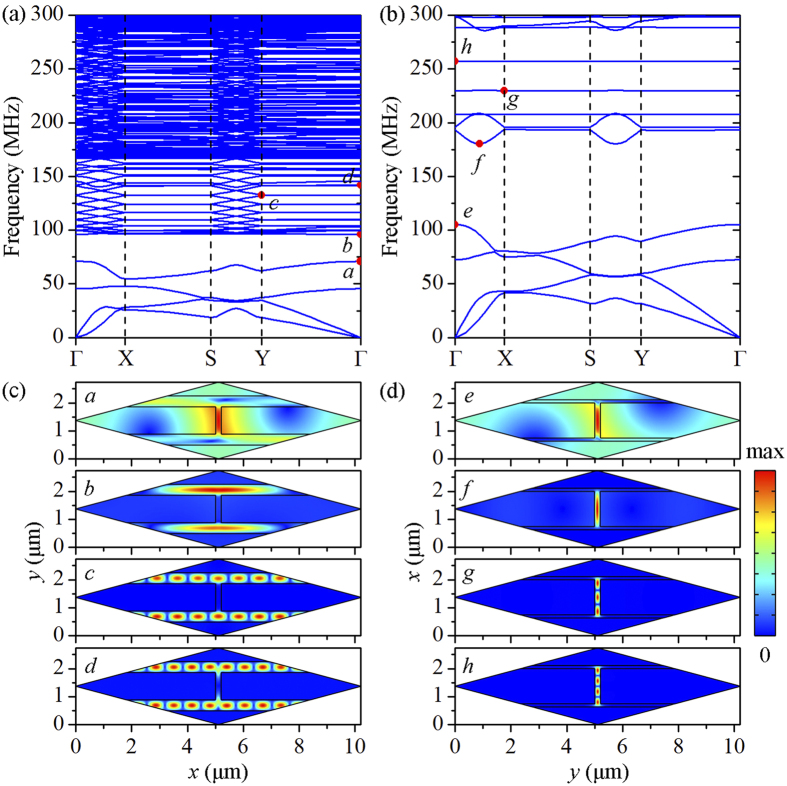
(**a**,**b**) The phonon dispersion relation of the lower layer and upper layer, respectively; (**c**,**d**) typical eigenmodes on the edges of band gaps in the lower layer and upper layer. The volume fractions of mineral platelets in the lower and upper layer are set to 0.70 and 0.90, respectively.

To gain insight into the physical mechanisms responsible for the broadband attenuation zones of the heterogeneous composite, phonon dispersion relations need to be constructed. The proposed heterogeneous composite as a whole is not periodic; however, each constituent layer indeed is a two-dimensional periodic structure. In this regard, the phonon dispersion relation of each layer can be calculated accordingly, as shown in Fig. 4(a,b). Notice that the elastic wave incident normally to the top surface of the heterogeneous composite corresponds to wave propagation along YΓ and ΓX directions for the lower and upper layer, respectively. The partial band gaps along YΓ and ΓX directions in the phonon dispersion relations agree well with the attenuation zones in the transmission spectrum of each constituent layer. By superimposing the partial band gaps along these two directions, we can obtain broadband attenuation zones, which are consistent with those in Fig. 2(a). This result further confirms our claim that the broadband wave attenuation capability of the heterogeneous composite is achieved by directly superimposing the attenuation zones in each constituent layer.

Here the band gaps corresponding to attenuation region I, II and III are taken as examples to reveal the physical mechanisms of band gap formation. As previously predicted, the first two band gaps in each layer are due to Bragg scattering^42^. This is also supported by the typical eigenmodes on the lower and upper edges of the band gaps, as shown in Fig. 4 (c,d). By contrast, some of the band gaps in the constituent layers, e.g., the ones pinned by point *c, d* and *g, h*, are believed to result from local resonances. This observation is supported by the distinctive feature that the band gaps are bounded by flat bands, indicating that zero group velocity exists and hence the energy flow is prohibited^6^,^7^,^9^,^43^. To give a better understanding of the local resonance effect, we plot in Fig. 4(c,d) the eigenmodes on the flat bands of the band gaps in lower and upper layers, respectively. The displacement field of each mode is totally localized in the soft organic matrix, preventing the elastic wave propagation. These characteristics indicate that the resulting band gaps are attributed to local resonances. Having determined the physical mechanisms of band gap formation in each layer, we are able to identify the mechanisms of superimposed attenuation region I, II and III in the heterogeneous composite, respectively. In short, the resulting broadband wave attenuation capability can be attributed to Bragg scattering and/or local resonances, depending on the mechanisms of the band gaps in each constituent layer.

### Effect of mineral platelet orientation and concentration on vibration mitigation

**Figure 5:**
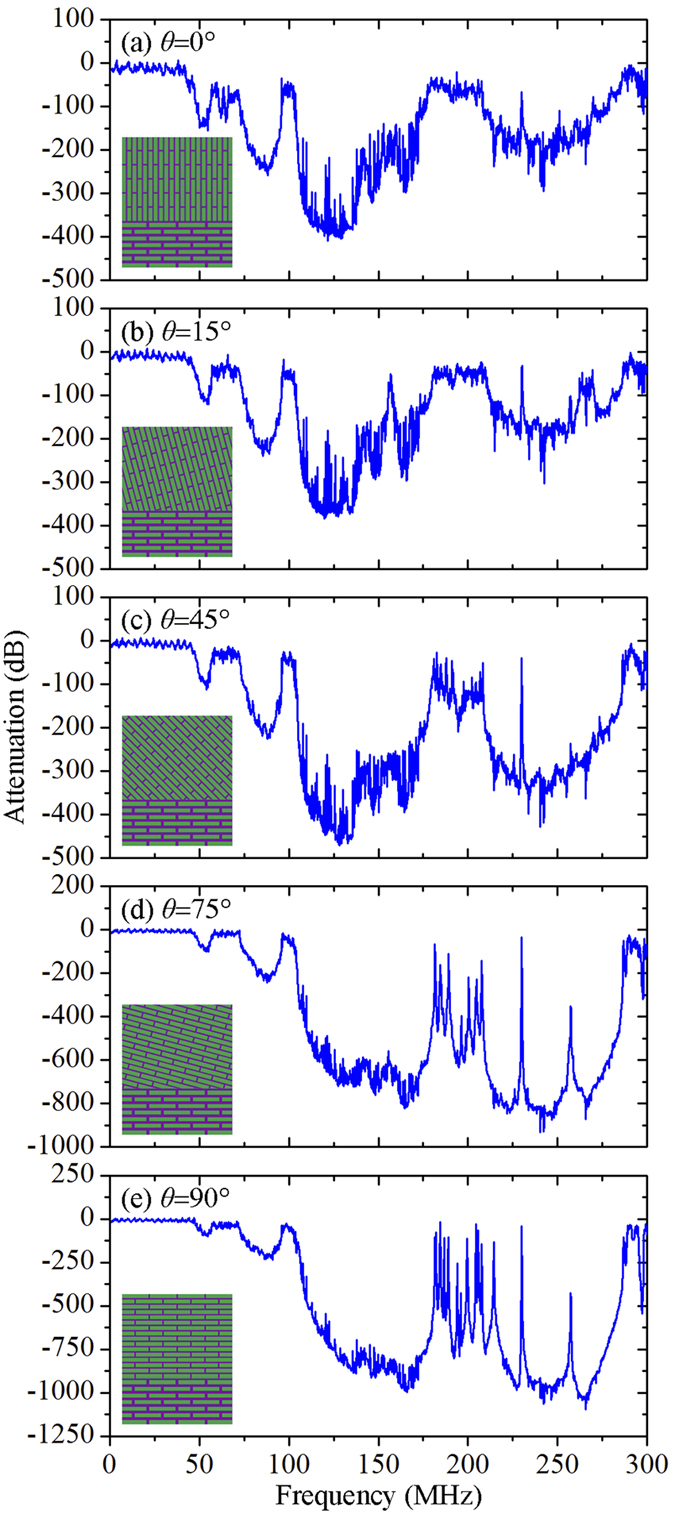
Effect of mineral platelet orientation in the upper layer on the wave attenuation. (**a**) *θ* = 0°, (**b**) *θ* = 15°, (**c**) *θ* = 45°, (**d**) *θ* = 75°, and (**e**) *θ* = 90°. The mineral platelets in the upper layered are rotated by *θ* in the counterclockwise direction. The volume fractions of mineral platelets in the lower layer and upper layer are set to 0.7 and 0.9, respectively.

Through millions of years’ evolution, biological materials have optimized their shapes to handle external multidirectional loading conditions, whereby the biological materials exhibit enhanced site-specific mechanical properties^25^,^30^,^31^,^32^,^44^,^45^. Importantly, this naturally widespread phenomenon has provided inspiration for researchers to produce heterogeneous composites with controlled reinforcement orientation and concentration^33^. We expect the proposed heterogeneous composites still exhibit broadband wave attenuation capability when the local orientation and concentration of the mineral platelets are rationally tailored to deal with external mechanical stimuli. To this end, we examine the effect of mineral platelet orientation and mineral platelet concentration on the transmission spectra of the proposed heterogeneous composites. Figure 5 shows the effect of mineral platelet orientation in the upper layer changing from 0° to 90° while remaining the same for the lower layer. Notice that the original broadband attenuation zones persist when the angle is rotated from 0° to 15°. When the alignment angle reaches 45°, additional attenuation zones arise around 195 MHz. Remarkably, multiple additional attenuation zones arise between 180 MHz and 208 MHz when the alignment angle reaches to 75° and 90°. It should be noted that the wave attenuation capability is progressively enhanced with the increase of the alignment angle from 0° to 90°. These results indicate that the proposed heterogeneous composites can maintain the broadband wave attenuation capability when the mineral platelet orientation is locally manipulated.

**Figure 6:**
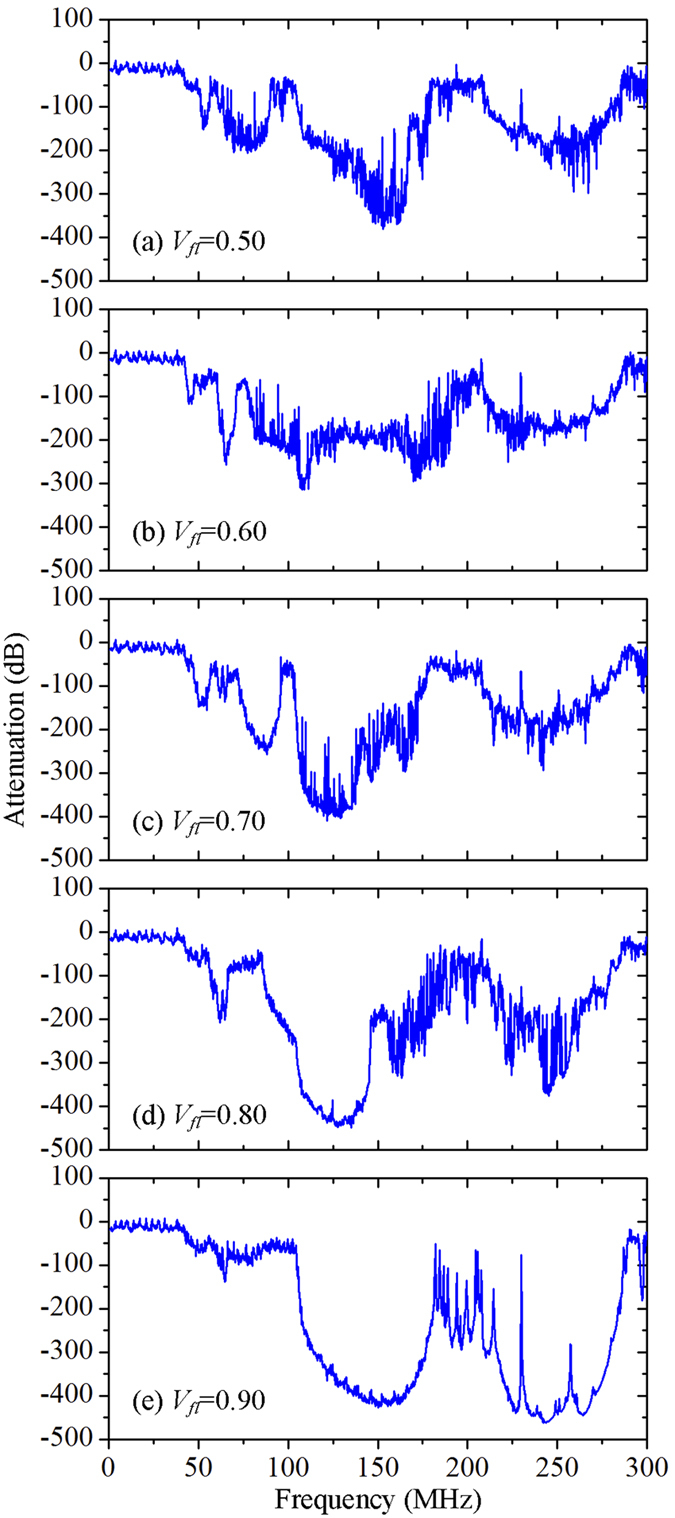
Effect of mineral platelet concentration in the lower layer (*V*_*fl*_) on the wave attenuation. (**a**)*V*_*fl*_ = 0.50, (**b**)*V*_*fl*_ = 0.60, (**c**)*V*_*fl*_ = 0.70, (**d**)*V*_*fl*_ = 0.80, and (**e**)*V*_*fl*_ = 0.90. The volume fraction of mineral platelets in the upper layer is set to 0.90.

We next explore the effect of local mineral platelet concentration of the proposed heterogeneous composites on the wave attenuation capability. The mineral platelet concentration in the lower layer is varied from 0.50 to 0.90, while that in upper layer remains 0.90. Figure 6 shows the effect of mineral platelet concentration in the lower layer on the wave attenuation capability. When the volume fraction is increased from 0.50 to 0.70, the bandwidths of the attenuation zones stay the same. However, the attenuation zones below 96 MHz tend to gradually disappear when the volume fraction is increased to 0.80 and 0.90. This is because the attenuation zones of the lower layer tend to shift towards higher frequency ranges and to mostly overlap with those in the upper layer, when the mineral platelet concentration in lower layer is increased to that in upper layer. This finding suggests that a contrast between mineral platelet concentrations of the two constituent layers is essential to maintain the broadband wave attenuation capability.

## Discussion

**Figure 7:**
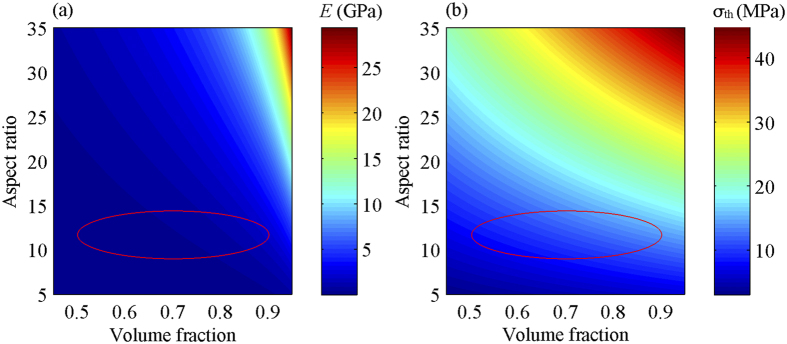
(**a**) Stiffness (*E*) and (**b**) strength (σ_th_) contour maps of each constituent layer of the proposed heterogeneous composite. The stiffness and strength as a function of aspect ratio and volume fraction of mineral platelets are predicted by the shear lag model^24^,^55^. The regions surrounded by the red ellipses in (**a**) and (**b**) indicate the stiffness and strength of the constituent layers of the proposed heterogeneous composites, respectively.

We have demonstrated the existence and robustness of broadband wave attenuation capability in the bio-inspired heterogeneous composites. We show that the broadband attenuation zones in the heterogeneous composites are achieved by directly superimposing the attenuation zones in each constituent layer. It should be pointed out that the constituent layers of the proposed heterogeneous composites in this work are based on the biological structural composites with high mechanical performance. We plot the stiffness and strength contour maps of the constituent layers as a function of the aspect ratio and the volume fraction of mineral platelets (Fig. 7). Note that each constituent layer of the proposed heterogeneous architecture has considerable stiffness and strength. In addition, the bio-inspired heterogeneous architecture with mineral platelets oriented out-of-plane in the upper layer and in-plane in the lower layer enables unusual combination of hardness, flexural modulus and fracture toughness^25^,^30^,^31^, ^32^, ^33^,^36^,^38^,^45^. However, a systemic investigation into the mechanical properties of the composite architecture is beyond the scope of this study. The broadband vibration mitigation capability combining the notable mechanical performance of the proposed heterogeneous architecture makes it particularly suitable for vibration mitigation and impact resistance in hostile environments, such as for deep water applications.

In general, a few approaches have been proposed to improve the wave attenuation capability of composite materials, including topological optimization^46^, introduction of fractal microstructures^47^, and external mechanical loading^48^,^49^. In our study, however, broadband attenuation zones are achieved by simply stacking two layers mimicking the heterogeneous architectures of structural biological materials. This flexible approach also endows the proposed heterogeneous composites with mechanical performance as compared to those achieved by other approaches. It should be pointed out that improved wave attenuation capability and enhanced mechanical properties can also be simultaneously achieved by introducing multilevel structural hierarchies in one-dimensional layered composites^50^. However, the resulting broadband attenuation zones are undermined and do not directly result from the superposition of those in each structural level, because the moduli are reduced and the structural periodicities are interrupted at higher levels. This phenomenon is more prominent in two-dimensional multilevel hierarchical composites.

We reveal that the attenuation zones in the proposed heterogeneous composites are attributed to Bragg scattering and/or local resonances. Besides dictating the band gaps of the heterogeneous composites, these physical mechanisms also contribute to the outstanding mechanical properties of structural biological composites^51^. Hypothetically, the multiple scattering and localization of elastic wave can control the interaction of elastic energy among locally high stress regions. As a result, more elastic energy will be dissipated by the soft organic matrix, and hence enhanced fracture toughness can be achieved. To some extent, this further supports our claim that broadband vibration mitigation and enhanced mechanical performance can be simultaneously achieved in the proposed heterogeneous composites. It should be noted that the material properties of the constituents, such as Young’s modulus and Poisson’s ratio, can have a great impact on the size of band gaps^42^. Therefore these parameters can be further utilized to tune the band gaps.

We also show that the original broadband attenuation zones will be maintained and additional attenuation zones will arise when the mineral platelet orientation in the upper layer is rationally manipulated. Importantly, a contrast between mineral platelet concentrations of each constituent layer is essential to generate broadband vibration mitigation. Indeed, manipulating local characteristics of heterogeneous architectures to handle externally mechanical challenges is prevalent in structural biological composites. For example, synthetic composites with locally tunable orientation and concentration of reinforcements have been recently reported^33^,^52^,^53^. By coating the reinforcements with minimal concentration of superparamagnetic nanoparticles, the orientation and concentration of the coated reinforcements can be remotely controlled using a ultralow magnetic field^33^,^52^,^53^. These progresses provide a flexible approach to precisely tailor the local characteristics of the proposed heterogeneous architecture, suggesting the possibility to design optimal multifunctional composites with improved wave attenuation capability and mechanical performance simultaneously.

In summary, we have reported broadband vibration mitigation capability in the bio-inspired heterogeneous composites that are based on the biological structural composites with high mechanical performance. Broadband wave attenuation capability is achieved by directly superimposing the attenuation zones in each constituent layer. Physical mechanisms, including Bragg scattering and local resonances responsible for the attenuation zones, have been identified by studying the phonon dispersion relation of each layer. The investigation into the effect of local characteristics of the heterogeneous composites on attenuation capability indicates that the broadband wave attenuation capability will be maintained, provided that a contrast between mineral platelet concentrations exists. The current study not only provides better understanding of the dynamic response of bio-inspired heterogeneous composites, but also opens new avenues to design optimal multifunctional composites for broadband vibration mitigation and impact resistance under mechanically challenging environmental conditions.

## Methods

### Calculation of transmission spectrum

**Figure 8:**
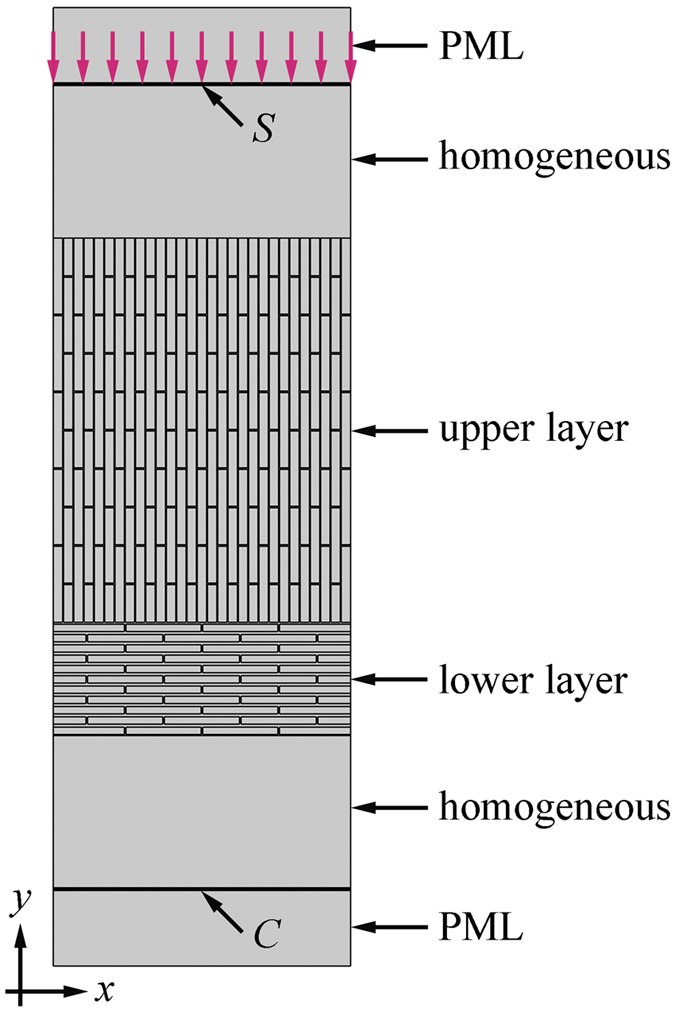
Finite element model for transmission spectra calculation. The model consists of three parts: the heterogeneous composites (14 × 5 unit cells in the upper layer and 4 × 5 unit cells in the lower layer), the homogeneous layers, and the perfectly matched layers. The material properties of the homogeneous layers and the perfectly matched layers are the same as those of pure organic matrix.

The transmission spectra were calculated by conducting frequency domain analyses. To model the elastic wave incident normally to the top surface of the heterogeneous composites, a harmonic vertical displacement with amplitude of 0.01 μm is applied on the line *S* (Fig. 8). Perfectly matched layers (PMLs) are applied at the two ends of the homogeneous parts to prevent reflections by the scattering waves from the domain boundaries^54^. The wave attenuation capability is defined as 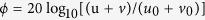, where *u* and *v* are the amplitudes of horizontal and vertical displacements collected on line *C,* respectively; *u*_*0*_ and *v*_*0*_ are the amplitudes of the applied horizontal and vertical displacements, respectively.

### Calculation of phonon dispersion relation

The phonon dispersion relations were obtained by solving wave equation for elastodynamics using the finite element method. The governing equation of elastic wave propagation in the heterogeneous composites can be written as


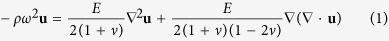


where **u** is the displacement vector, and *ω* is the angular frequency. *E, v*, and *ρ* are the Young’s modulus, the Poisson’s ratio, and the density of each constituent phase, respectively. According to Bloch’s theorem, Floquet periodic boundary conditions are applied at the boundaries of the unit cell such that


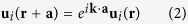


where **r** is the location vector, **a** is the lattice translation vector, and **k** is the wave vector.

The governing equation (1) combining with the boundary condition, equation (2), leads to the standard eigenvalue problem:


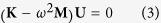


where **U** is the assembled displacement vector, and **K** and **M** are the global stiffness and mass matrices assembled using standard finite element analysis procedure, respectively. The unit cell is discretized using 6-node triangular elements. Eq. (3) is numerically solved by imposing two components of wave vectors and hence yields the corresponding eigenfrequencies. The phonon dispersion relations are obtained by scanning the wave vectors along the edges of the first Brillouin zone. (Fig. 1(e)).

## Additional Information

**How to cite this article**: Chen, Y. and Wang, L. Bio-inspired heterogeneous composites for broadband vibration mitigation. *Sci. Rep.* **5**, 17865; doi: 10.1038/srep17865 (2015).

## References

[b1] Graczykowski B. *et al.* Phonon dispersion in hypersonic two-dimensional phononic crystal membranes. Phys. Rev. B 91, 075414 (2015).

[b2] Lee J. H. *et al.* 25th anniversary article: Ordered polymer structures for the engineering of photons and phonons. Adv. Mater. 26, 532–569 (2014). 24338738 10.1002/adma.201303456 PMC4227607

[b3] Maldovan M. Sound and heat revolutions in phononics. Nature 503, 209–217 (2013). 24226887 10.1038/nature12608

[b4] Kushwaha M. & Halevi P. Band-gap engineering in periodic elastic composites. Appl. Phys. Lett. 64, 1085–1087 (1994).

[b5] Khelif A., Aoubiza B., Mohammadi S., Adibi A. & Laude V. Complete band gaps in two-dimensional phononic crystal slabs. Phys. Rev. E 74, 046610 (2006). 10.1103/PhysRevE.74.046610 17155195

[b6] Achaoui Y., Khelif A., Benchabane S., Robert L. & Laude V. Experimental observation of locally-resonant and Bragg band gaps for surface guided waves in a phononic crystal of pillars. Phys. Rev. B 83, 104201 (2011).

[b7] Liu Z. *et al.* Locally resonant sonic materials. Science 289, 1734–1736 (2000). 10976063 10.1126/science.289.5485.1734

[b8] Ho K. M., Cheng C. K., Yang Z., Zhang X. & Sheng P. Broadband locally resonant sonic shields. Appl. Phys. Lett. 83, 5566–5568 (2003).

[b9] Goffaux C. & Sánchez-Dehesa J. Two-dimensional phononic crystals studied using a variational method: Application to lattices of locally resonant materials. Phys. Rev. B 67, 144301 (2003).

[b10] Chen Y. & Wang L. Periodic co-continuous acoustic metamaterials with overlapping locally resonant and Bragg band gaps. Appl. Phys. Lett. 105, 191907 (2014).

[b11] Vasseur J., Djafari-Rouhani B., Dobrzynski L., Kushwaha M. & Halevi P. Complete acoustic band gaps in periodic fibre reinforced composite materials: the carbon/epoxy composite and some metallic systems. J. Phys.-Condens. Mat. 6, 8759 (1994).

[b12] Kushwaha M., Halevi P., Martinez G., Dobrzynski L. & Djafari-Rouhani B. Theory of acoustic band structure of periodic elastic composites. Phys. Rev. B 49, 2313 (1994). 10.1103/physrevb.49.2313 10011063

[b13] Chen Y., Yao H. & Wang L. Acoustic band gaps of three-dimensional periodic polymer cellular solids with cubic symmetry. J. Appl. Phys. 114, 043521 (2013).

[b14] Zhang S., Xia C. & Fang N. Broadband acoustic cloak for ultrasound waves. Phys. Rev. Lett. 106, 024301 (2011). 21405230 10.1103/PhysRevLett.106.024301

[b15] Maldovan M. Narrow low-frequency spectrum and heat management by thermocrystals. Phys. Rev. Lett. 110, 025902 (2013). 23383916 10.1103/PhysRevLett.110.025902

[b16] Zen N., Puurtinen T. A., Isotalo T. J., Chaudhuri S. & Maasilta I. J. Engineering thermal conductance using a two-dimensional phononic crystal. Nat. Commun. 5, 3435 (2014). 24647049 10.1038/ncomms4435 PMC3973070

[b17] Gonella S., To A. C. & Liu W. K. Interplay between phononic bandgaps and piezoelectric microstructures for energy harvesting. J. Mech. Phys. Solids. 57, 621–633 (2009).

[b18] Lv H., Tian X., Wang M. Y. & Li D. Vibration energy harvesting using a phononic crystal with point defect states. Appl. Phys. Lett. 102, 034103 (2013).

[b19] Jiang H. & Wang Y. Phononic glass: A robust acoustic-absorption material. J. Acoust. Soc. Am. 132, 694–699 (2012). 22894191 10.1121/1.4730922

[b20] Baravelli E. & Ruzzene M. Internally resonating lattices for bandgap generation and low-frequency vibration control. J. Sound. Vib. 332, 6562–6579 (2013).

[b21] Wegst U. G., Bai H., Saiz E., Tomsia A. P. & Ritchie R. O. Bioinspired structural materials. Nat. Mater. 14, 23–36 (2014). 25344782 10.1038/nmat4089

[b22] Chen P. Y. *et al.* Structure and mechanical properties of selected biological materials. J. Mech. Behav. Biomed. 1, 208–226 (2008). 10.1016/j.jmbbm.2008.02.003 19627786

[b23] Meyers M. A., Chen P. Y., Lin A. Y. M. & Seki Y. Biological materials: structure and mechanical properties. Prog. Mater. Sci. 53, 1–206 (2008). 10.1016/j.jmbbm.2008.02.003 19627786

[b24] Wang L. & Boyce M. C. Bioinspired structural material exhibiting post-yield lateral expansion and volumetric energy dissipation during tension. Adv. Funct. Mater. 20, 3025–3030 (2010).

[b25] Ortiz C. & Boyce M. C. Bioinspired structural materials. Science 319, 1053–1054 (2008). 18292331 10.1126/science.1154295

[b26] Yao H., Xie Z., He C. & Dao M. Fracture mode control: a bio-inspired strategy to combat catastrophic damage. Sci. Rep. 5, 8011 (2015). 25619564 10.1038/srep08011 PMC4306140

[b27] Li H., Yue Y., Han X. & Li X. Plastic deformation enabled energy dissipation in a bionanowire structured armor. Nano Lett. 14, 2578–2583 (2014). 24745628 10.1021/nl500379t

[b28] Huang Z., Pan Z., Li H., Wei Q. & Li X. Hidden energy dissipation mechanism in nacre. J. Mater. Res. 29, 1573–1578 (2014).

[b29] Barthelat F. & Zhu D. A novel biomimetic material duplicating the structure and mechanics of natural nacre. J. Mater. Res. 26, 1203–1215 (2011).

[b30] Qi H., Bruet B., Palmer J., Ortiz C. & Boyce M. C. [Micromechanics and macromechanics of the tensile deformation of nacre]. *Mechanics of biological tissues*. [ Holzapfel G. A. & Ogden R. W. (ed.)] [189–203] (Springer-Verlag, Berlin Heidelberg, 2005).

[b31] Studart A. R. Biological and bioinspired composites with spatially tunable heterogeneous architectures. Adv. Funct. Mater. 23, 4423–4436 (2013).

[b32] Studart A. R. Towards high-performance bioinspired composites. Adv. Mater. 24, 5024–5044 (2012). 22791358 10.1002/adma.201201471

[b33] Erb R. M., Libanori R., Rothfuchs N. & Studart A. R. Composites reinforced in three dimensions by using low magnetic fields. Science 335, 199–204 (2012). 22246772 10.1126/science.1210822

[b34] Chai H., Lee J. J. W., Constantino P. J., Lucas P. W. & Lawn B. R. Remarkable resilience of teeth. P. Natl. Acad. Sci. USA 106, 7289–7293 (2009). 10.1073/pnas.0902466106 PMC2678632 19365079

[b35] Song J., Ortiz C. & Boyce M. C. Threat-protection mechanics of an armored fish. J. Mech. Behav. Biomed. 4, 699–712 (2011). 10.1016/j.jmbbm.2010.11.011 21565718

[b36] Wang L., Song J., Ortiz C. & Boyce M. C. Anisotropic design of a multilayered biological exoskeleton. J. Mater. Res. 24, 3477–3494 (2009).

[b37] Bruet B. J., Song J., Boyce M. C. & Ortiz C. Materials design principles of ancient fish armour. Nat. Mater. 7, 748–756 (2008). 18660814 10.1038/nmat2231

[b38] Han L., Wang L., Song J., Boyce M. C. & Ortiz C. Direct quantification of the mechanical anisotropy and fracture of an individual exoskeleton layer via uniaxial compression of micropillars. Nano Lett. 11, 3868–3874 (2011). 21755939 10.1021/nl201968u

[b39] Espinosa H. D. *et al.* Tablet-level origin of toughening in abalone shells and translation to synthetic composite materials. Nat. Commun. 2, 173 (2011). 21285951 10.1038/ncomms1172

[b40] Aksay L. *et al.* Biomimetic Pathways for Assembling Inorganic Thin Films. Science 273, 892–898 (1996). 8688064 10.1126/science.273.5277.892

[b41] Fleischli F. D., Dietiker M., Borgia C. & Spolenak R. The influence of internal length scales on mechanical properties in natural nanocomposites: a comparative study on inner layers of seashells. Acta. Biomater. 4, 1694–1706 (2008). 18617447 10.1016/j.actbio.2008.05.029

[b42] Chen Y. & Wang L. Tunable band gaps in bio-inspired periodic composites with nacre-like microstructure J. Appl. Phys. 116, 063506 (2014).

[b43] Wang P., Casadei F., Shan S., Weaver J. C. & Bertoldi K. Harnessing buckling to design tunable locally resonant acoustic metamaterials. Phys. Rev. Lett. 113, 014301 (2014). 25032927 10.1103/PhysRevLett.113.014301

[b44] Barthelat F., Li C. M., Comi C. & Espinosa H. D. Mechanical properties of nacre constituents and their impact on mechanical performance. J. Mater. Res. 21, 1977–1986 (2006).

[b45] Fratzl P. & Weinkamer R. Nature’s hierarchical materials. Prog. Mater. Sci. 52, 1263–1334 (2007).

[b46] Sigmund O. & Jensen J. S. Systematic design of phononic band–gap materials and structures by topology optimization. Philos. T. Roy. Soc. A 361, 1001–1019 (2003). 10.1098/rsta.2003.1177 12804226

[b47] Castineira-Ibánez S., Romero-García V., Sánchez-Pérez J. & Garcia-Raffi L. Overlapping of acoustic bandgaps using fractal geometries. Europhys. Lett. 92, 24007 (2010).

[b48] Wang L. & Bertoldi K. Mechanically tunable phononic band gaps in three-dimensional periodic elastomeric structures. Int. J. Solids. Struct. 49, 2881–2885 (2012).

[b49] Bertoldi K. & Boyce M. Mechanically triggered transformations of phononic band gaps in periodic elastomeric structures. Phys. Rev. B 77, 052105 (2008).

[b50] Zhang P. & To A. C. Broadband wave filtering of bioinspired hierarchical phononic crystal. Appl. Phys. Lett. 102, 121910 (2013).

[b51] Davies B. *et al.* Hypothesis: Bones toughness arises from the suppression of elastic waves. Sci. Rep. 4, 7538 (2014). 25518898 10.1038/srep07538 PMC4269876

[b52] Erb R. M., Sander J. S., Grisch R. & Studart A. R. Self-shaping composites with programmable bioinspired microstructures. Nat. Commun. 4, 1712 (2013). 23591879 10.1038/ncomms2666

[b53] Libanori R. *et al.* Stretchable heterogeneous composites with extreme mechanical gradients. Nat. Commun. 3, 1265 (2012). 23232395 10.1038/ncomms2281

[b54] Khelif A., Achaoui Y., Benchabane S., Laude V. & Aoubiza B. Locally resonant surface acoustic wave band gaps in a two-dimensional phononic crystal of pillars on a surface. Phys. Rev. B 81, 214303 (2010).

[b55] Ji B. & Gao H. Mechanical properties of nanostructure of biological materials. J. Mech. Phys. Solids. 52, 1963–1990 (2004).

